# Foraging supply chains: Investigating disaster for improved food provisioning

**DOI:** 10.1007/s13280-025-02205-w

**Published:** 2025-06-18

**Authors:** Hana Trollman, Sandeep Jagtap, Sonia D. Tamakloe, Frank Trollman

**Affiliations:** 1 University of Leicester School of Business, Leicester, UK; 2 Division of Engineering Logistics, Lund University, Lund, Sweden; 3 Sustainable Manufacturing Systems Centre, Cranfield University, Cranfield, UK; 4 Trauma and Orthopaedics Department, University Hospitals of Derby and Burton, Derby, UK

**Keywords:** Ecological embeddedness, Food provisioning, Food supply chain, Foraging theory, Minimum viable ecosystem, Sustainable development

## Abstract

Disasters such as COVID-19 and the Russia–Ukraine war are drawing attention to the provisioning of food during crises. The main concern has been quickly establishing a stable food supply. However, climate change and public health concerns are shifting attention to the critical gap in identifying the minimal considerations that would adequately address ecological disaster food provisioning. A meta-ethnography of 16 disasters in 12 different countries is employed to identify the activities and their supporting strategies that provide benefits to existing actors within food networks. Analysis suggests that public health, resilience, and sustainability stand to benefit from the identified practices. A conceptual model of an ecologically embedded minimum viable ecosystem for disaster food provisioning is proposed. Exemplar applications are provided for Tigray, Gaza, and Ukraine. The findings may be applied to disaster settings for the development of policy for culturally sensitive, equitable, and nutritious food provisioning strategies.

## Introduction

This research investigates ecologically embedded disaster food provisioning for improved regional sustainability, resilience, and public health. The common impacts of such disasters encompass damaged infrastructure, compromised local food production, and obstructed food imports (e.g., Jagtap et al. 2024). Although ensuring regional sustainability, ecological safety, and agri-food supply chain viability under conditions of war is beginning to be contemplated in terms of a cloud-based enterprise resource planning system (Kopishynska et al. 2023), there is a lack of a conceptual understanding for operationalization. Conceptual understanding of an ecosystem approach supports more advanced problem-solving alongside transferability to new contexts of disaster food provisioning globally.

Addressing disaster food provisioning in a regional context is critical for economic, environmental, and social reasons. From an economic perspective, food security is linked to state sovereignty through the development of regional economies (Yerlan et al. 2023). For example, the Russia–Ukraine war has had significant impacts on food security due to effects on agri-food supply chains both for the nations at war and globally (Jagtap et al. 2022, 2024; Rudolfsen et al. 2024). From an environmental perspective, an increase in violent conflicts has long been predicted due to growing shortages of water, forest resources, fisheries, and arable land (Homer-Dixon 1994). Environmental change and resource scarcity align with the identification of precursors of violent conflict by the Intergovernmental Panel on Climate Change: declining water resources, reduced food security, and potential population migrations (IPCC 2007). The resulting social impacts from such scenarios may be forced reliance on humanitarian food supplies and/or (re-) localized food sourcing (Ying and Egermann 2024).

Current approaches to conceptualizing impacts from an ecological perspective include war/warfare ecology and disaster ecology. However, these are broad approaches with few practical insights for disaster food provisioning. For example, war ecology identifies reduced consumption as a reactive adjustment to major disruption to fossil fuel supply (Charbonnier 2022), but disruptions to food supplies and food markets are not detailed at the supply chain level. A comprehensive and holistic approach to disaster food provisioning needs to consider all potential sources of food provisions, potential beneficiaries, and sustainability impacts. We argue that ecological embeddedness (as defined by ecosystem sustainability, ecological relationships, and nutritional ecology) remains an afterthought when it comes to crisis food provisioning (e.g., Cattivelli and Rusciano 2020) despite global concerns related to public health and environmental issues (climate change, pollution, biodiversity loss).

In a viable ecosystem, there is a balance between population and food availability. Food supply is viable when its existence and functionality is maintained in a practical and effective manner. Minimum viable ecosystem (MVE) has been defined as the smallest configuration of activities and actors that creates value such that existing participants are retained and new actors are integrated, especially those with unique capabilities, to create an optimal solution (Adner 2003). Food availability during crises relies on civilian, military, and/or humanitarian supply chains that may obtain various and irregular donations from local and global sources, reflecting foraging behavior. This research conceptualizes MVE in the context of foraging theory to extend its application to food supply chains and provide a novel perspective on disaster food provisioning.

To identify the requirements of an MVE for food provisioning subject to war-like conditions, particularly with desirable constraints such as ecological embeddedness (Trollman et al. 2022), the following question is posed:

What are the key constituents of an ecologically embedded MVE for disaster food provisioning?

To answer this question, a meta-ethnographic synthesis is conducted to inform conceptual understanding of food provisioning (Britten et al. 2002; Sattar et al. 2021). This approach is adopted because meta-ethnographies offer the potential for theoretical innovation (Urrieta and Noblit 2018). Optimal Foraging Theory (OFT) is utilized because sustainable procurement (e.g., Grob and Benn 2014; Xu et al. 2022) and sustainable supply chain theory (e.g., Suryawanshi et al. 2021; Wang et al. 2023) lack holistic perspectives that encompass ecological embeddedness. Consequently, this work will be of interest to both practitioners of food resource management and academicians for contributing to the knowledge base on disaster food provisioning.

This research makes two contributions. The case-study-based meta-ethnography collects rare and globally diverse instances of disaster food insecurity impacts at community level to reveal effective strategies for equitable food provisioning that support sustainability and resilience. The meta-ethnography then leads to conceptualization based on MVE. Below we briefly review ecological embeddedness and present the theoretical framework. Then the meta-ethnographic synthesis is described in detail. Finally, a discussion of findings and limitations is presented.

## Literature review

In this literature review, due to the volume of literature on armed conflict and its impacts on agricultural production, consumption, and nutrition, the focus is limited to ecological perspectives. Comprehensive reviews relating armed conflicts and food insecurity are noted by Rudolfsen et al. (2024). Armed conflict can impact agricultural production by disrupting the supply of water, fertilizer, veterinary medicines, etc., with immediate and/or long-term effects on food supply chains. Although disaster food provisioning is by many considered solely in terms of international and/or non-regional assistance, an ecological approach necessitates not only the consideration of the ecological impacts of such sourcing (e.g., pollution), but also the preservation and support of viable local sources.

Below we justify the argument that ecosystem sustainability, ecological relationships, and nutritional ecology are the necessary trifecta for improved outcomes in disaster food provisioning by highlighting existing gaps in the literature. The theoretical lens is described in detail.

### Ecological embeddedness

Fundamentally, ecological embeddedness is a locally responsive strategy to local ecosystems that has been developed in support of ecosystem sustainability. The construct of ecological embeddedness was first introduced by Whiteman and Cooper (2002) in their study of a Cree tallyman in northern Quebec, encompassing the extent to which there is personal identification with the land; and adherence to beliefs of ecological respect, reciprocity and caretaking. Added conceptual clarity to the construct was provided by Morris and Kirwan (2011) by exploring alternative food networks to find the necessity of reflecting relationships between economic actors and the underlying ecology of production such that economic activity is influenced to produce a benefit to both. Landrum and Ohsowski (2018) connected ecological embeddedness to more mature levels of corporate sustainability worldviews. Trollman and Colwill (2021) extended ecological embeddedness to strategy formulation in manufacturing, also connecting ecological embeddedness with legitimation in circular economy (Trollman et al. 2020).

However, ecological embeddedness has not been fully considered at the level of supply chains. Supply chains have been described as socio-environmental ecosystems (Walker et al. 2004), meaning that actors constantly reorganize and adapt to their environment (Gruner and Power 2017; Legenvre et al. 2022). Trollman et al. (2022) extended consideration of ecological embeddedness to supply chains in the limited context of a single manufacturer, introducing blockchain for waste as a potential solution for supply chain by-product valorization alongside considerations of quality and safety arising from traceability and visibility in a coffee supply chain. The utilization of by-products and reduction of supply chain waste is important in disaster situations, which may be supported by digital technology, for improved utilization of resources (Bounie et al. 2020). Importantly, for ecological embeddedness, there should be benefits for all actors of the supply chains and the environment that comprise the relevant socio-environmental ecosystem in the disaster situation.

In spite of the benefits of ecological embeddedness, networks of ecologically embedded supply chains have been underexplored. Ecological reasoning has been proposed to provide a link between ecological sensemaking and ecological embeddedness to help organizations ensure that their employees and the consumers of their products understand the impact of organizational actions on ecological processes (Hannah et al. 2023). Network structures in Italy have been examined in the context of illegal construction with future work planning to investigate a territory rather than individual companies (Troisi et al. 2023). Importantly, ecological embeddedness has yet to be applied to networks of supply chains under disaster conditions—namely identifying the requisite supporting ecological relationships.

Having established that ecosystem sustainability and ecological relationships have not been fully considered for networks of supply chains, the following section advances OFT as the missing link to incorporate nutritional ecology as a necessary third element for ecologically embedded disaster food provisioning.

### Foraging theory

In modern society, food supply chains are the predominant means of satisfying dietary needs for most people. Under war-like conditions, even modern food supply chains need to forage. Foraging supply chains may obtain unplanned and/or impromptu food in the form of donations to humanitarian organizations and/or (re-)localized food production as opposed to pre-established contractual relations for procurement. This research seeks to contribute to the understanding of foraging theory in modern society under disaster conditions by investigating foraging supply chains constrained by ecological embeddedness, extending the idea of foraging factories (Factories that Forage 2017).

A key consideration of extending foraging theory to modern supply chains is the incorporation of nutritional ecology. Although the fields of OFT and nutritional ecology developed independently, their integration has influenced many areas of biology and biomedical science (Raubenheimer and Simpson 2018). OFT connects foraging with microeconomics, decision theory, and operations research. The connection between OFT, nutritional ecology, and disaster situations is exhibited through the constraints placed on the quantity and quality of available food with the related dietary challenge of identifying a combination of deficits and surpluses that minimize the related cost.

Foraging theory has been historically applied to human foragers in relation to cultural selection and transmission due to the cultural variation evident in foraging (Smith et al. 1983). Table 1 illustrates the major decision categories of OFT for human hunter-gatherers (Smith et al. 1983). However, this conceptualization needs to be updated to reflect application to modern food supply chains. Below, we indicate the known effects that disaster has on each of the decision categories of OFT from Table 1, demonstrating that current literature and practice do not adequately reflect ecological embeddedness of foraging theory.

**Table 1:** Main elements of OFT in hunter-gatherer society

Strategic goal	Decision category	Domain of choice	Example constraints
Optimal set of resources for exploitation	Diet breadth	Types to harvest	Search and pursuit abilities of forager
Optimal set of resources for exploitation	Diet breadth with nutrient constraints	Type and quantity to harvest	Nutrient requirement; prey abundance
Optimal set of exploitable habitats	Patch choice	Which set of patches to visit	Habitat richness; travel time between patches
Optimal time pattern allocation to alternatives	Time allocation	Time spent foraging in each alternative	Depletion rates
Optimal foraging group size	Foraging group size	Size of foraging groups	Rules for harvest division
Optimal home base location	Settlement pattern	Settlement location for each foraging unit	Effects of cooperation and competition

*Diet breadth:* There is inherent flexibility in decision making because human nutritional requirements of protein, carbohydrates, and fats comprise less than half the nutritional requirements of calories overall, allowing for humans to thrive on radically different diets (Ryan-Harshman and Aldoori 2006). Furthermore, very high activity levels substantially increase total calorie needs, while having comparatively little effect on necessary carbohydrates, proteins, and fats (Yon and Johnson 2005). This versatility allows different cultures and individual preferences to be supported by menus that differ not only in specifics, but of the broadest sense of food categories. Under disaster conditions, this allows for significant substitution of diets at the biological level. Unfortunately, non-biological barriers to food substitution exist, and historically food waste has increased when humans have been asked to use substituted sources of nutrition, even in disaster situations (Trollman et al. 2021a, 2021b).

*Diet breadth with nutrient constraints:* Disaster conditions complicate foraging in terms of nutritional requirements, putting strain on menus and forcing substitution. Severe social disruptions such as war and other disasters affect food choice, disrupting healthy dietary patterns, which can continue past the initial disruption (Trollman et al. 2021a, 2021b; Munialo and Mellor 2023). Physical activity and stress increase calorie needs, and humans in disaster conditions frequently have increased need for nutrition relative to their needs during times of stability. It is ironically true that many scenarios that reduce available food also increase the need for food, creating a virtual shortfall that is larger in terms of its effect on human health than would be expected from the raw numbers of projected shortfall (Nutrition humanitarian needs analysis guidance 2020). Additionally, even during active conflicts, most military personnel are generally not active warfighters, and this creates a need for diversity of menu quantity (also to avoid food waste) between administrative and field staff, whose calorie needs will differ dramatically. Current practices include humanitarian daily rations which are packages that are typically air-dropped into disaster areas, and the preference of the European Union to help vulnerable people access food by giving them money when there is enough food in shops and markets. Meals, Ready to Eat (MREs) are the military staple for those in the field, but these are not intended as a replacement for fresh foods.

*Patch choice and time allocation:* Disaster conditions complicate foraging in terms of logistical difficulties. This may include interruptions in supply chains, human displacement, and loss of means of food storage and preparation. The supply networks that may be affected include both civilian and military supply chains which may be interrelated when food is supplied to the disaster-affected region. In other words, military supply chains may support civilian relief efforts and civilian suppliers may be integrated into military supply chains to provide food for personnel engaged in relief and/or war efforts. Harnessing traceability at individual item level in humanitarian food supply chains is only just being explored, promising sustainability improvements such as the ability to assess the carbon footprint of commodity transport (Tantillo 2023).

*Foraging group size:* Determining the appropriate number and type of food supply chains to serve a disaster location is important for positive impact on the served populations. While humans are biologically capable of obtaining their nutritional needs from many sources, extra barriers exist that limit the effectiveness of humanitarian relief that is not culturally appropriate. History has numerous incidents of secondary disasters where food sent to afflicted populations was partially or wholly unusable due to lack of relevant tools, knowledge, or cultural background (Bruin Political Review 2022).

*Settlement pattern:* Foraging food supply chains may access relocalized food production as one strategy that has the potential to provide healthy food at affordable prices (Mikulić et al. 2023). However, food safety and food quality are of particular concern to decision makers when food supply is disrupted due to food perishability and associated risks (e.g., Jagtap et al. 2022). Based on the nature of the foraged food, its preparation and consumption are skilled activities that utilize tools and sources of energy (often heat for cooking and cool temperatures for storage), which may or may not be available at specific locations in addition to having varying environmental impacts.

**Fig. 1:**
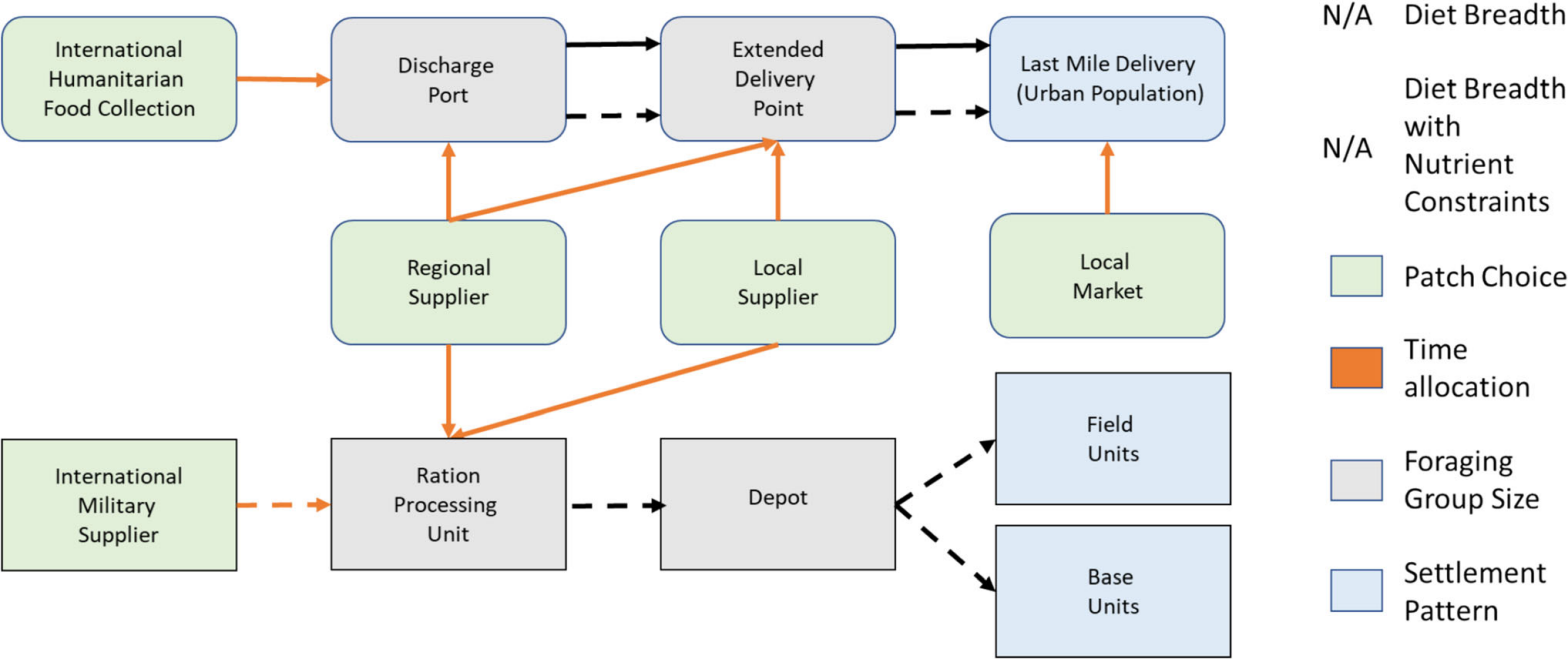
High-level application of OFT to existing food provisioning in a disaster region. Military resources may be used to deliver humanitarian supplies as indicated by the upper dashed lines. Diet breadth is inadequately considered (noted as N/A) with proposed models making assumptions such as every beneficiary (of a certain type in a humanitarian food supply chain) receives the same food basket, no beneficiary goes unfed, the modeled nutrition measure is a simple average across all nutrients, and that the supply chain network is fixed (e.g., Peters et al. 2021)

Figure 1 illustrates the food supply chains that may be present under disaster conditions in the context of OFT based on combing the work of Peters et al. (2021) on humanitarian assistance and Mohammed et al. (2023) on military supply chains, noting the inadequacy of considerations of diet breadth. Sustainability considerations are generally not reflected in current approaches.

In summary, under war-like conditions, foraging intersects with food provisioning. However, ecologically embedded responses to food shortages via supply chains are not well understood and consequently have inadequate implementation leading to poor nutrition, waste, and unsustainable use of resources.

### Theoretical lens

War ecology is a field of study that encompasses preparations for war, the war (violent conflict itself), and post-war activities (Machlis and Hanson 2008). This research addresses situations aligned with the war stage, specifically food supply which intersects with civilian rationing, materiel supplies (military equipment and products), infrastructure, and governance. The anthropocentric nature of the driving forces implies interdisciplinarity and highly coupled biophysical and socioeconomic systems.

Early literature on the principles of ecology as they pertain to the impact of war neglected food supply chains and the roles of organisms in maintaining the whole system (Lanier-Graham 1993). Volatility, uncertainty, complexity, and ambiguity (VUCA) describe the dynamic environment that disaster supply chains operate under with success being based on collaboration, coordination, sovereignty, and equality in distributing resources (Katina and Gheorghe 2023). Humanitarian food supply chains are neither well understood by researchers nor adequately coordinated in terms of their provisioning of fresh food in sudden-onset disasters (Giedelmann-L et al. 2022). There is also a need for military supply chains to be agile and robust for multi-agent coordination in anticipation of undesirable states (Kaddoussi et al. 2011).

The analysis of supply chains has been extended to the ecosystem level in terms of viability (Ivanov and Dolgui 2020). The interconnected supply chains that are the subject of such analysis are open systems with structural dynamics, conceptualized in terms of their viability. The building of viable supply chain designs and viability of different ecosystems (e.g., agriculture, healthcare) have emerged as new research areas (Ivanov and Dolgui 2020). However, the aforementioned research does not consider the role of a minimum configuration.

Ali and Gossaye (2023) are notable for combining supply chain resilience, agility, and sustainability in a single study on supply chain performance using viability—a novel approach in supply chain literature, but well-known in ecological modeling and cybernetics. However, Ali and Gossaye (2023) consider viable supply chains of large manufacturers that survive long-term global disruptions as opposed to a broader network affected by regional disaster.

The minimum viable product (MVP) is a powerful approach in the new product development process (Sundmaeker 2016; Wongsaichia et al. 2024). The MVP is a product with just sufficient features to attract early-adopter customers, enabling feedback for future development. An MVP is immediately introduced to the market to test hypotheses and gain knowledge for rapid identification of a workable option (Deloitte Consulting LLP 2015).

The identification of minimum configurations is important for rapid response to disaster conditions. Ecosystem innovation has expanded MVP to MVE (Lewrick et al. 2018) such that global supply chains are viewed as potential MVEs supporting embedded sustainability (Liao et al. 2023). The concept of MVE has also been considered in the context of new technology in the agri-food sector (Rampone et al. 2023). However, there has been a lack of theoretical development for supply chain MVE, particularly for food provisioning under disaster conditions. Consequently, MVE is a suitable lens from which to examine foraging supply chains.

In summary, this research on disaster food provisioning applies foraging theory through the lens of MVE to contribute to the understanding of the complex interactions that support fair and adequate dietary health delivery alongside minimal environmental impact.

## Methodology

Meta-ethnography is an alternative approach to conducting syntheses of qualitative research that involves induction and interpretation. Meta-ethnographies offer the potential for theoretical innovation (Urrieta and Noblit 2018). A meta-ethnographic synthesis approach was utilized to aid in the conceptual understanding of MVE for the ecologically embedded provisioning of food in a region subject to conditions of disaster to obtain higher order interpretation compared to conventional narrative literature reviews (Britten et al. 2002; Sattar et al. 2021). The seven steps of Noblit and Hare (1988) were employed to conduct the meta-ethnography.

Meta-ethnographies have been used to study healthy eating strategies (Gillies et al. 2021), personal and community values behind sustainable food consumption (Lamarque, et al. 2023), children’s and women’s nutritional health and well-being in the context of food insecurity in Europe (Bell et al. 2023a, 2023b), and food in the prison environment (Woods-Brown et al. 2023). However, food network actors have yet to be investigated using meta-ethnography.

Initially, a synthesis of the topic was judged necessary due to the lack of conceptual and theoretical understanding of MVEs, given that there is a large and growing body of qualitative research in the respective areas of humanitarian, military, and civilian food supply chains during crises. The authors constitute a research team with different approaches, opinions, and the key skills to conduct the meta-ethnography.

As the synthesis is intended to incorporate findings from humanitarian, civilian (relocalization), and military food supply chains, representative papers were selected from each of these areas. Furthermore, relevant papers from disaster relief were included. It is important to note the following assumptions: the papers selected are commensurable (concepts are transferable across settings), given that there is the importance of including studies in disparate settings (including different countries) (Britten et al. 2002).

**Fig. 2:**
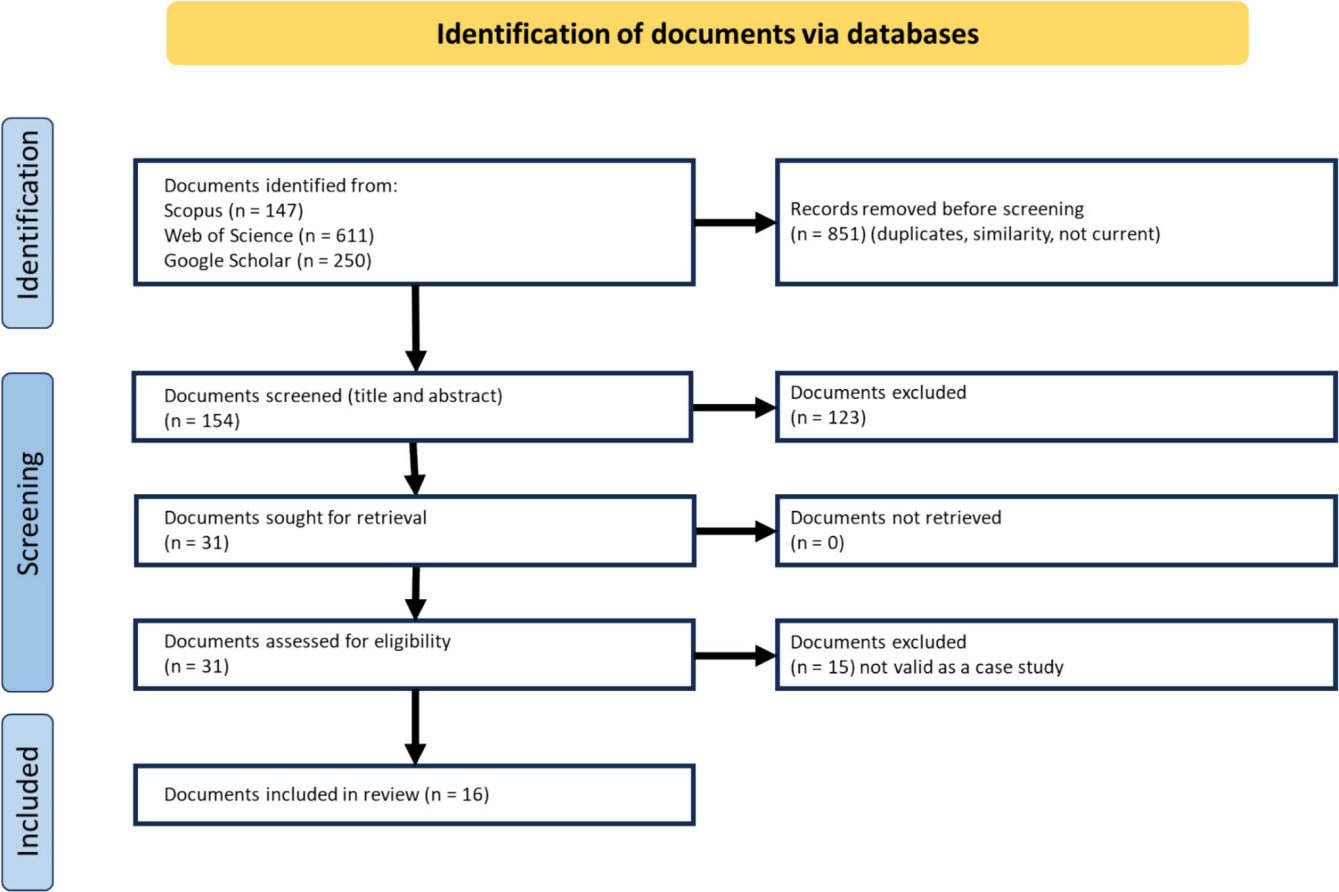
A PRISMA flow diagram showing documents identified, screened, and included (Page et al. 2021)

Searches were conducted on Scopus on 20 December 2023. The search terms used were guided by the focus of this research which is on food supply chains under conditions of disaster. Specifically, searches were conducted using “food supply chain” AND “military” (12 results, 2 case studies), “food supply chain” and “humanitarian” (19 results, 2 case studies), “food supply chain” AND “relocalization” OR “relocalisation” (16 results, 1 case study), “food supply chain” AND “disaster” (100 results, 12 case studies but 1 removed due to similarity). Figure 2 shows the PRISMA numbers.

Confirmatory searchers were conducted to ensure that appropriate diversity in the case studies was obtained. On February 20, 2023, a search was conducted using Web of Science under “All Databases”. This search found “food supply chain” and “military” (73 results), “food supply chain” and “humanitarian” (159 results), “food supply chain” and “relocalization” (16 results)/“relocalisation” (3 results), “food supply chain” and “disaster” (360 results).

Google Scholar search restricted to 2020–2024 (February 20, 2024) was undertaken to capture any additional relevant cases starting from the COVID-19 period. The COVID-19 period reflects the beginning of a recognized paradigm shift in supply chains, highlighting their fragility. This restriction was applied only to the confirmatory Google Scholar search due to the large number of results for some categories. The searches under “food supply chain” and “military” identified about 2,930 results; “food supply chain” and “humanitarian” about 4,020 results; “food supply chain” and “relocalization” about 145 results/“relocalisation” about 128 results; “food supply chain” and “disaster” about 6,500 results. Titles were read for the first 5 pages (10 results per page).

The 16 selected case studies provided the necessary balance between manageability and sufficient studies to avoid underdeveloped theories/concepts, ensuring rich descriptive data suitable for meta-ethnography. Rigor, credibility, and relevance of each source were ranked by three authors to exclude those with major flaws based on the Qualitative Assessment and Review Instrument (JBU-QARI—a ten-item checklist: jbi.global/critical-appraisal-tools accessed 22 December 2023). The number of cases selected aligned with a current review of meta-ethnographies that found a sample size of 17 ± 8 studies on average (Soundy and Heneghan 2022). As is evident, the scope of meta-ethnography is often more restricted than many narrative reviews. The 16 case studies included in the meta-ethnography are shown in Table 2.

**Table 2:** Sources for the meta-ethnography

No.	References	Title	Nature of case study	Research methods
1	Dymyt and Wincewicz-Bosy (2023)	Fresh Food Deliveries to Military Units During COVID-19 Pandemic	Military unit (unspecified)	Literature review, document analysis, reports, participant observation, semi-structured interviews, case study, mapping, and analysis
2	Winecewicz-Bosy et al. (2022)	Military Food Supply Chain during the COVID-19 Pandemic	Military unit (Poland)	Single case study, semi-structured interviews, supply chain mapping, process mapping
3	Muhialdin et al. (2021)	Traditional foodstuffs and household food security in a time of crisis	Mosul, Iraq	Case study, ethnography, in-depth, semi-structured interviews, literature review, thematic analysis, coding
4	Barsing et al. (2018)	Cross-docking center location in a supply chain network: a social network analysis approach	Fast-food chain company (India)	Social network analysis
5	Gava et al. (2018)	Linking sustainability with geographical proximity in food supply chains. An indicator selection framework	Bread supply chains—one global and two local (Italy)	Case study, interviews, literature review
6	Bakker et al. (2023)	School or parking lot? Selecting locations for points of distribution in urban disasters	Supermarket closures in Berlin, Germany	Case study
7	Munialo and Mellor (2023)	A review of the impact of social disruptions on food security and food choice	COVID-19, conflict-affected zones: South Sudan, Russian invasion of Ukraine, etc.	Review with multiple cases
8	Gil et al. (2023)	Strategic approach to food system resiliency from community-based initiatives during the Covid-19 pandemic	Eight community-based initiatives that either rebuilt or started up a food supply chain during COVID-19 outbreak peaks in Medellín, Colombia	Case study (three stages), semi-structured interviews, stakeholder mapping technique, constant comparison method, open coding
9	Umar and Wilson (2023)	Inherent and adaptive resilience of logistics operations in food supply chains	Two supply chain wide case studies in two different South Asian regions (Pakistan)	Explorative case study methodology, nonprobability sampling, purposive sampling, semi-structured interviews, informal conversations, field notes, logistic documents, coding (NVivo 11 software)
10	Giedelmann-L et al. (2022)	System dynamics approach for food inventory policy assessment in a humanitarian supply chain	Mocoa, Colombia, South America	System dynamics model (simulation)
11	Reis et al. (2022)	Working through disaster risk management to support regional food resilience: a case study in north-eastern Australia	Cairns, north-eastern Australia	Literature review, case study, stakeholder workshops, stakeholder survey (online and telephone interviews)
12	Umar and Wilson (2021)	Supply chain resilience: unleashing the power of collaboration in disaster management	Two different South Asian regions	Multiple case study, purposeful sampling, face-to-face interviews, observations, informal conversations, field notes, documents, reports, review of secondary sources, coding (NVivo 11)
13	Ekren et al. (2021)	Lateral inventory share-based models for IoT-enabled E-commerce sustainable food supply networks	Online grocery network (B2B) with single-echelon food supply chain	Simulation (OptQuest tool in ARENA 16.0 simulation package)
14	Marusak et al. (2021)	Resilient regional food supply chains and rethinking the way forward: Key takeaways from the COVID-19 pandemic	Seven case studies of regionalized food supply chains in Texas and Iowa	Multiple case study, interviews
15	Chodur et al. (2018)	Assessing food system vulnerabilities: A fault tree modeling approach	Two well-characterized events: Baltimore City food system during Winter Storm Jonas of 2016, agricultural production data from California during the 2013–2017 drought	Fault tree using a top-down approach guided by expertise, extant literature, and 36 stakeholder interviews
16	Smith ansd Lawrence (2014)	Flooding and food security: A case study of community resilience in Rockhampton	Rockhampton, State of Queensland	Qualitative case study; analysis of policy, media, and government literature; qualitative, semi-structured interviews, coding

The studies were read and reread by two of the authors to identify first- and second-order constructs. The data was extracted verbatim to preserve the original terminology used by the primary authors. The information collected included the study sample, data collection methods, data analysis methods, study outcomes, and study conclusions. The two authors then looked across the studies for common and recurring concepts as part of an iterative process. Descriptive labels for the themes were identified to form new categories.

The studies were then translated into one another. In this phase, each concept from each paper was compared with all the other papers to check whether the commonality existed or not. This process highlighted similarities and differences between the concepts and metaphors so that third-order constructs were identified. The key concepts were discussed among three of the authors to produce collaborative interpretations.

The identification of similarities and differences employed reciprocal and refutational synthesis followed by line of argument synthesis. Third-order constructs, themes, and global themes were identified. The process was iterative such that the authors read and reread the identified studies to create a final data table.

The eMERGE reporting guidance (France et al. 2019) and PRISMA guidelines (Page et al. 2021) were used to produce the summary of findings, strengths, limitations, and reflexivity, as well as recommendations and conclusions. All authors reviewed and contributed to the final synthesis.

### Validation

First-phase validation was prima facie validation by an author not involved in the initial development of first- and second-order constructs, and included collaboration in refining the conceptual model.

Second-phase validation was undertaken by a fourth author independently and without prior knowledge of the identified constructs. This further validation employed the five criteria constituting a good synthesis—cogency, economy, apparency, range and credibility (Noblit and Hare 1988).

The second-phase validation led to recommendations that were addressed before the presentation of results. By systematically evaluating the data against the five meta-ethnographic criteria above, confirmation was obtained of the reliability, validity, originality, and high-quality of the data.

## Results

The line of argument synthesis is presented below which includes both comparable and oppositional data interpretations (Sattar et al. 2021). A line of argument synthesis translates the accounts that interpret different aspects of the same phenomenon under study such that a whole is produced that is greater than the sum of individual parts (Noblit and Hare 1988). Contributions to theory and literature are integrated into the synthesis.

Second-order constructs were identified to facilitate the reciprocal and refutational translation as described in the Methodology. Six second-order constructs were identified: nutrition, sustainability, relationships, intermediaries, alternative actors/solutions, and structures, as described below.

### Reciprocal translation identified (accounts are directly comparable)

The second-order construct “nutrition” featured prominently as the case studies were mainly concerned with supplying nutritious food to human populations. The human populations were described as educated, rich, poor, with/without children, elderly, vulnerable, and soldiers with/without health concerns. The food supplied was described variously as local, fresh, and/or traditional. Food could be perishable or long shelf-life, low/high/poor/relaxed/ quality, safe, and/or culturally suitable. Food that was of better quality was sourced locally or regionally whereas food from global supply chains was generally regarded as being of poor nutritional quality. The second-order construct “nutrition” aligns with the strategic goals of foraging theory related to locating resources to provide dietary breadth. The sources may be constrained by dietary restrictions of specific members of the population, the availability of cold chains to preserve freshness, cost, and public health considerations.

“Sustainability” was also common across studies, being represented in terms of business continuity, efficiency (including energy efficiency, administrative efficiency), waste (especially food waste due to perishability), pollution/emissions, biodiversity, and ecological/environmental considerations. “Intermediaries” featured as facilitating sustainability in terms of business continuity (described as a safety buffer for military supply chains). Sustainability intersects with three strategic goals of foraging theory: distribution and foraging structure for improved efficiency and minimization of pollution/emissions/waste; and time allocation to ensure the availability and sustainability of resources.

“Relationships” were both contractual as in the case of military supply chains and civilian supply chains contracting with logistics providers, and non-contractual or informal such as cases of sharing logistics functions, resources (including food), and inventory. When intermediaries or alternative actors were involved in relationships, an emphasis was placed on trust. Relationships are part of the selection of sources in foraging theory where sources are interpreted as suppliers instead of patch choice.

“Alternative solutions”, generally facilitated by alternative actors, included alternative modes of transport, alternative financing, alternative networks, and alternative conflict resolution in support of sustainability given the insufficiency of traditional methods under the given situation. Alternative solutions extend foraging theory from suppliers and logistics to a broader network including finance and judiciary solutions.

“Structures” were considered in terms of distribution: warehouse location, distribution points, and cross-docking (inbound goods are directly transferred to outbound transportation with no or minimal storage time); strategy: centralized/decentralized/parallel; collaboration: horizontal/vertical; supply chains: long/short; networks: network capital, cohesive networks (eliminate dependence on a single supply chain node to minimize risk of failure), supply chain networks, social networks, alternative food networks, new/community-based emergency food networks, dominant (modern) food networks, communication networks, network resources (e.g., vehicles, storage facilities, labor), food supply networks, e-grocery networks, sustainable food networks; models (conceptualizations of food system): fault tree modeling (for conceptual assessment of food system resilience) and lateral inventory share-based models for e-grocery networks (where customer orders are sent to customers directly without physical transfer to another online grocery at the same supply chain level). Fast delivery was identified as a solution supporting sustainability regardless of the structure involved. Structures are specific to modern food supply chains and so are not explicitly considered under foraging theory.

### Refutational translation identified (accounts are oppositional)

The process of comparing oppositional accounts identified elements related to modern supply chains not explicitly considered under foraging theory. However, these elements help to define the MVE as described below.

Although intermediaries were seen as valued contributors for business continuity, they were also identified as being less common in centralized structures and short (regional) supply chains, leading to greater efficiency. Therefore, intermediaries in these cases would not be aligned with sustainability. The inclusion of intermediaries in the MVE thus needs to be informed by the sustainability considerations of foraging theory.

Alternative solutions were not advanced by military supply chains even though there was reliance on intermediaries to spread risk. Intermediaries were able to participate in military supply chain tenders and would therefore be subject to contracts. The role of the military was both in supplying civilian populations and itself (military units), but considerations related to prioritization or interoperability were lacking. Reliance on military supply chains would mainly affect selection of sources and foraging structure, which may be detrimental to creating an MVE for reasons of excluding potentially beneficial sources and inefficiencies which may lead to unsustainable solutions.

Although packaging was noted in relation to transformation activities, with military units relying on sub-suppliers of packaging, standardized packaging for improved business sustainability was a feature of civilian food supply chains in South Asia. The importance of packaging relates to both MVP and MVE and has not been given the prominence it deserves in terms of maintaining freshness, which may improve nutrient quality and enhance sustainability due to reduced waste.

The strategic structure was mainly considered at the operational level. Strategic-level considerations were not present except in the overarching aims of military and humanitarian supply chains, respectively. Centralized and decentralized structures were advanced based on the more pressing advantage: humanitarian supply chains were considered to benefit more from centralized structures, but parallel supply chains were proposed for improved sustainability. Similarly, centralized civilian food hubs could be prone to collapsing entire networks in case of failure. Horizontal collaborative structure facilitated coopetitive behavior (shared resources and common solutions) supporting business sustainability. Vertical collaboration also supported business sustainability, but information sharing was key as opposed to horizontal collaboration under which certain types of information sharing could compromise market differentiation. Considerations of structure are therefore critical to MVE.

### Line of argument synthesis

The third-order constructs identified in accordance with the Methodology were Existing Actor Activities, Supporting Strategies, Benefits to Existing Actors, and New Actors and Their Benefits. These themes align with MVE as the smallest configuration of activities and actors to create value that retains existing actors and integrates new actors to create an optimal solution. Below is the argument for the identification of the related global themes.

Similarity in Existing Actor Activities was exhibited in that actors all sought to supply food (whether nutritious or not) to human populations, predominantly in urban areas. The question arising is, given the value universally associated in the case studies with local or regional supply chains, would there be more sense in supplying both non-urban and non-human populations with greater priority to maintain a functioning ecosystem? The main motivation of food supply chain actors is to preserve the consumers of their products, otherwise, food supply chain actors may lose their customers; however, local or regional supply chains are reliant on animals and geographically dispersed producers.

Another similarity across sources was the aim to sustain the entire population, including the weak and infirm. Under normal business conditions and akin to predators in ecosystems (eliminating the weak and infirm), business would not universally provide this type of support—organizations such as charities would aid those unable to afford products. This draws attention to the transitional nature of the relationships forged to provide what is intended to be temporary aid (although situations like war, drought and plague may have a protracted course) informing the global theme of Transitional Operational Linkages. Consequently, the overarching global theme was identified as Diversity in Human Sustenance where diversity refers to not only the food supply being both local/regional and global, but also in terms of the diversity of the human population supplied.

The third-order construct identified as Supporting Strategies included a wide variety of operational level strategies to try to ensure the viability of food supply. These fell into two different categories: those focused on improving efficiency usually with associated environmental benefits, and those employing relationships to support continuity. The third-order construct Benefits to Existing Actors varied across studies, but generally provided benefits to supply chain actors and the environment in line with ecological embeddedness. Commonality was found in food quality and food safety; however, not all cases were able to supply nutritious food; some improved efficiency in specific cases such as small batches (cross-docking), and some were able to prevent panic buying, avoid bottlenecks, minimize days of food shortage, reduce average inventory, improve customer service, decrease need for large safety stocks, and/or make food affordable for vulnerable populations. Both above third order constructs informed the global theme Material and Process Efficiency.

The third-order construct New Actors and Their Benefits inductively postulated the addition of new actors to support the aims of the existing actors. The new actors were predominantly intermediaries/alternative actors/facilitators of a transitory nature. The question arising is if these transitional products and services could become permanently integrated for improved future resilience? As noted above, these transitory actors enable operations, but a strategic level for the ecosystem is lacking to incorporate them fully and permanently. Consequently, the Transitional Operational Linkages global theme was further supported.

**Fig. 3:**
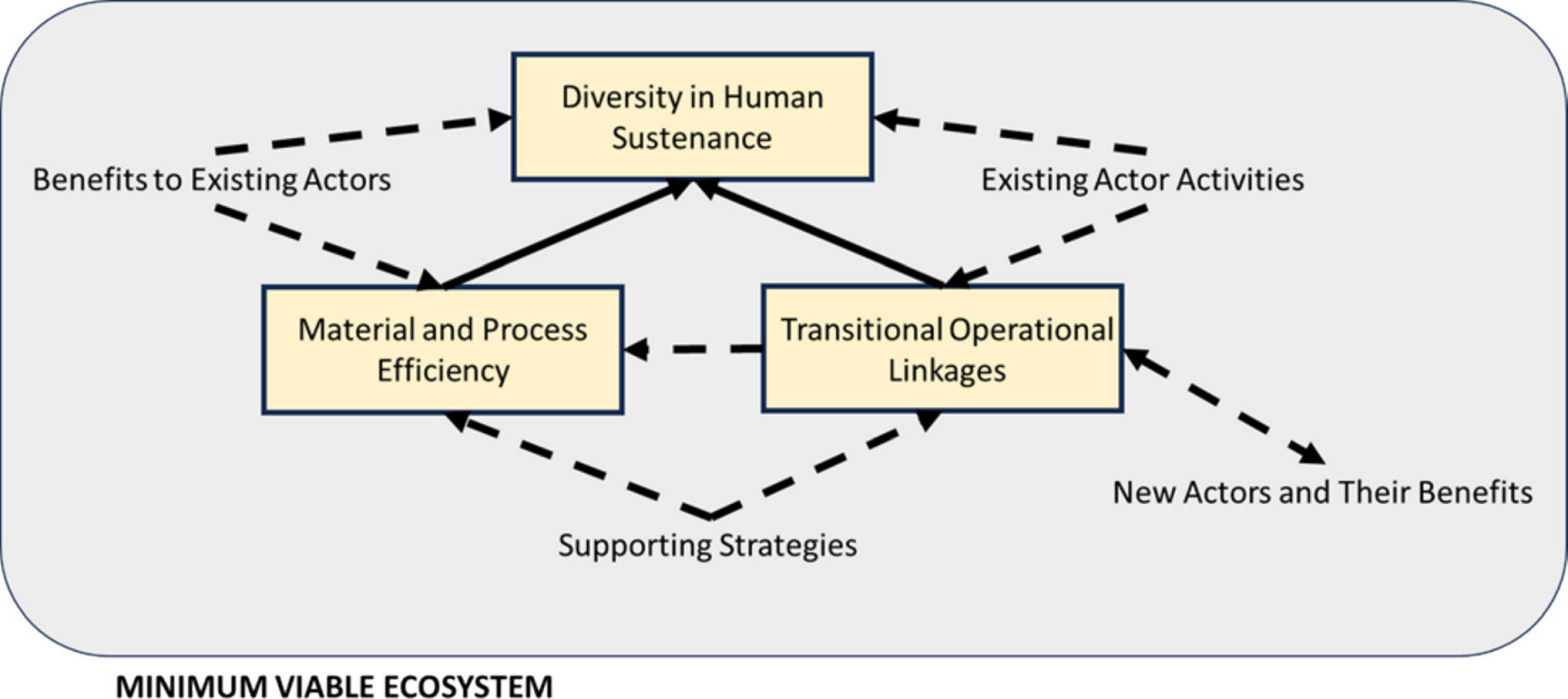
Conceptual model of a MVE for food provisioning under war-like conditions. Existing actors include humanitarian food supply chains, military supply chains, long and short supply chains. Solid arrows represent strong connections, whereas dashed arrows are weaker links (not always present)

In summary, the four themes were aligned with the MVE definition: Existing Actor Activities, Supporting Strategies, Benefits to Existing Actors, and New Actors and their Benefits. The three global themes that were identified from the four themes were Diversity in Human Sustenance; Material and Process Efficiency; and Transitional Operational Linkages. Figure 3 presents the conceptual model based on the line of argument synthesis.

## Discussion

The meta-ethnography identified three global themes: Diversity in Human Sustenance; Material and Process Efficiency; and Transitional Operational Linkages. The conceptual model (Fig. 3) indicates that the latter two are subordinate to the achievement of the first. The framework of adaptation strategies for viable supply chains during the COVID-19 pandemic has four major dimensions: scalability, repurposing or process flexibility, substitution or structural reconfiguration, and intertwining (Ivanov 2021). The conceptual model for MVE of food supply chains clearly highlights the centrality of human sustenance, and that material and process efficiency are more aligned with sustainable supply chains (Ahi and Searcy 2013). As noted by Gualandris et al. (2024), supply chain management research is still missing approaches for economically sustainable supply chains that regenerate social–ecological systems. Furthermore, as noted by Shakibaei et al. (2024), there has been limited consideration of sustainability dimensions with a predominantly single criterion approach to supply chain disruptions. Consequently, this research contributes to a better understanding of the processes supporting viable social–ecological systems under conditions of disaster.

### Theoretical contributions

Initially, this discussion will explore theory related to MVE in terms of the following three concepts: nutritional ecology, ecosystem sustainability, and ecological relationships. This approach is motivated by the comparison of ecosystems and supply chains, indicating that from a practical perspective, supply chains and ecosystems can either compete or complement each other because non-contractual and contractual relations in supply chains and ecosystems serve different purposes. Both supply chains and ecosystems can coexist and compete when neither of the relationships are dominant, and can complement each other when the environment is complex and rapidly evolving (Legenvre et al. 2022). Also, sustainable supply chains may be developed by mimicking natural ecosystems (Gruner and Power 2017).

Nutritional ecology is an interdisciplinary science examining all components of the food chain and evaluating their effects from the perspectives of health, environment, society, and economy. Although nutrition sciences are dominated by health considerations and food quality, this more holistic perspective of nutritional ecology is necessary to avoid ecological harm and achieve nutritional security for the world population (Claus 2003). Nutritional ecology envisages equal distribution of food resources, food choice for a healthy diet, and sustainable environment impact (Nutritional Ecology International Center n.d.). As such, it is aligned with foraging theory. Consequently, nutritional ecology intersects with the two global themes identified for MVE: Diversity in Human Sustenance and Material and Process Efficiency. However, a search on Scopus (Article title, Abstract, Keywords) for “nutritional ecology” and “supply chain” yielded two results (15 November 2024), indicating that this could be an area for future investigation. Both of these papers assess the level of health, socioeconomic and environmental sustainability of market garden vegetable production, trade and processing in Africa, but not under disaster conditions (Assinou and Kpotchou 2024a, 2024b). The nutritional ecology of obesity that is increasingly prevalent in human food chains considering rising atmospheric CO_2_ levels has been explored (Raubenheimer et al. 2015), but the importance of supplying nutritious food during times of crisis, when possibly the need is greatest due to the additional stresses of the situation, should be addressed in the context of nutritional ecology.

Ecosystem sustainability comprises consideration of ecological integrity, social equity, economic efficiency (of the business ecosystem), and intergenerational equity (Lu et al. 2015; Bova 2022). Consequently, ecosystem ecology intersects all three of the global themes identified: Diversity in Human Sustenance in terms of intergenerational equity; Material and Process Efficiency in terms of ecological integrity and economic efficiency; and Transitional Operational Linkages in terms of social equity. However, ecosystem sustainability has not been considered for supply chains in great depth, although as in the case of this research, regional markets and short food supply chains have been identified as resilient alternatives to connect urban populations with territorial agroecological production (Rover et al. 2020). The main challenge in terms of ecosystem health remains the effective integration of ecological understanding with a holistic view of socioeconomic, biophysical, biogeochemical, and public policy dimensions (Lu et al. 2015).

There is little understanding of how the concepts of ecological relationships could be applied in the context of disaster food provisioning. Ecological relationships include interspecific interaction, competition, predation, symbiosis, mutualism, commensalism, and parasitism. Interspecific interaction can be related to vertical collaboration in the supply chain. This research uncovered coopetitive behavior (simultaneous cooperation and competition) in horizontal collaboration of supply chains under war-like conditions. Both vertical and horizontal collaboration were identified as resulting from mutual dependence and/or mutual interests. Predatory relationships exist in business, but are unlikely to advance mutual interests: for example, predatory pricing or certain types of corporate takeovers. Drastic changes such as corporate mergers or natural disasters threaten survival (sustainability). Although competition exists as a means of achieving survival related to improved access to resources, the difference between corporate mergers and natural disasters is that in the case of the latter, the renewal rate of the immediate environment is respected as opposed to depleting natural resources required for production (Gruner and Power 2017). Fundamentally, based on the principle of interdependence, competition should not extend to the environment (Gruner and Power 2017). For beneficiaries such as the general population, parasitism may be a temporary means of achieving survival (sustainability) by being supplied with food during times of war. This may be considered parasitic for subtracting resources from the supply chain without providing any directly or indirectly valuable resource for the supply chain or its ecosystem (Bova 2022); or possibly in a commensalism relationship, depending on the circumstances. In terms of ecological embeddedness, mutualism (a special type of symbiosis) is the ideal ongoing form of relationship as it brings benefits to all actors and is best for long-term sustainability (Bova 2022). Ecological relationships relate to the global theme of Transitional Operational Linkages.

Finally, there is opportunity for improvement within each of the three global themes to advance theory as part of future work. Transitional Operational Linkages could be developed into longer-term relationships supported by mutualism. Material and Process Efficiency could be expanded into effectiveness. Diversity in Human Sustenance could be developed into sustenance and regeneration of the entire ecosystem. To achieve these aims, the requirement to incorporate additional actors into the MVE was identified.

Next, foraging food supply chains are considered. The ability of food supply chains to forage is constrained by the ecosystem they are located in and the populations they are intended to serve. Table 3 revisits the OFT of Table 1 to relate the major decision categories with analogues for foraging food supply chains supported by the cases of the meta-ethnography, thereby operationalizing the conceptual model in Fig. 3. In some circumstances, especially in developed countries where resources are not so much an issue, the entire population may be equitably and sufficiently supported. However, in many other cases, inequalities will exist that may be either systemic or characteristic of an individual or group. In these cases, Table 3 may guide an optimal overarching plan to mitigate inequalities and preserve the MVE.

**Table 3:** Foraging analogues for human foragers and food supply chains

Common strategic goal	Decision category		Domain of choice		Example constraints
Both	Human	Supply chain	Human	Supply chain	Supply chain
Optimal set of resources	Diet breadth	Procurement	Types to harvest	Perishable and/or nonperishable food	Cold chain Public health Special diets
Optimal set of resources	Diet breadth with nutrient constraints	Procurement	Type and quantity to harvest	Quantities of perishable and/or nonperishable foods	Cold chain Public health Special diets Procurement costs
Optimal set of sources	Patch choice	Suppliers	Which set of patches to visit	Local and/or relocalized and/or global	Military/humanitarian
Optimal time allocation	Time allocation	Collaboration	Time spent foraging in each alternative	Horizontal and/or vertical	Availability and sustainability
Optimal foraging structure	Foraging group size	Logistics (distribution network)	Size of foraging groups	Centralized and/or decentralized	Military/humanitarian supply chains
Optimal distribution	Settlement pattern	Population served (urban, rural)	Settlement location for each foraging unit	Last mile delivery: distribution point and/or individual homes	Vulnerable populations

### Practical contributions

In terms of the MVE, global supply chains, particularly in the case of humanitarian supply chains, remain significant contributors to maintaining Diversity in Human Sustenance. This means it is also not possible to draw a physical boundary around the ecosystem that would not be planetary in nature. However, the locality principle does not imply that supply chains need to be completely constrained (Gruner and Power 2017). The efforts of policy-makers preparing for disasters should be directed at improving their MVE such that it is more resilient and able to sustain itself for as long as possible without resorting to uncertainties of outside aid. In some regions, this may be more feasible than others given food-specific issues such as seasonality and sources (e.g., open field cultivation, greenhouses) of crop production. Yet these barriers are not insurmountable with advances in, for example, vertical farming (Gruner et al. 2013), which is still considered to hold great promise in spite of recent business failures (Taylor 2025). In practice, during the COVID-19 pandemic, produce from a ‘shipping container’ research vertical farm was donated to help feed the homeless in Nottinghamshire, UK (Rogers 2019; Zagnat 2020), providing more nutritious natural crops with far bigger yields and faster harvest cycles.

Practitioners will benefit from examining the strategies employed/modeled in the case studies to determine whether implementation could improve their MVEs based on the principle of heterogeneity (Gruner and Power 2017). Suggested strategies include:

1. Parallel centralized and decentralized provisioning (Smith and Lawrence 2014; Marusak et al. 2021; Giedelmann-L et al. 2022)
2. Distribution points: location (Bakker et al. 2023) and fault identification at provisioning points (Chodur et al. 2018)
3. Cross-docking for perishable food (Barsing et al. 2018; Marusak et al. 2021)
4. Vertical and horizontal supply chain collaboration (Smith and Lawrence 2014; Ekren et al. 2021; Marusak et al. 2021; Umar and Wilson 2021)

However, there is a lack of tools to aid holistic decision making that incorporate ecological considerations in times of disaster such as those suggested for non-crisis situations, for example, a circularity indicator tool for ecological embeddedness (Trollman et al. 2021a, 2021b). Below we describe three current exemplars for application of OFT from a MVE perspective.

### Applications

#### Tigray, Ethiopia

The war in Tigray (Northern Ethiopia) started in 2020 and brought devastation to smallholder farmers and food security. Prior to the war, more than 80% of the rural population in Tigray was primarily engaged in subsistence agriculture. These rural areas suffered damage such as the burning of crops, destruction of farm equipment and irrigational structures, and slaughter of livestock. Humanitarian food aid was cut-off or hijacked. In some areas, people were forced to move out of larger settlements (cities, towns, and villages) to escape to remote gorges, rivers, caves, and gullies (Meaza et al. 2024). An assessment of empirical evidence of war damage on smallholder agriculture in Tigray (Manaye et al. 2023) indicates that 53% of the crop disruption occurred in one month (November) which is the annual harvest time for key crops.

Applying the conceptual model of MVE, Material and Process Efficiency needs to be established as a priority (Fig. 3). The reason for this is the rural nature of the population alongside the difficulty of supplying humanitarian food aid. Sharing and cooperation among the rural population need to be established to make optimal use of remaining resources to improve food security. Operationalization via OFT requires addressing the issues of procurement and suppliers due to the settlement pattern (rural) (Table 3), employing local knowledge and strategies given accessible and available resources. Clearly, attempts to deliver food aid need to continue as well, but rehabilitation of infrastructure and provision of agricultural inputs are longer-term support for the next annual harvest.

#### Gaza

Israel is intentionally depriving Gaza of food, water, and energy in addition to obstructing humanitarian aid from outside sources (Human Rights Watch 2023). Many farms have been destroyed, and food price inflation is rampant. There is a lack of safe water that can be used for drinking and food cooking, and a lack of cooking gas has meant that people burn wood and rubbish instead. Chemical warfare is leaving air, soil, and water pollution, as well as biodiversity loss and other potential ecological damages. Desperate measures of the populace include consuming animal feed and weeds to stave off hunger due to the destruction of infrastructure for food supply chains (Hassoun et al. 2024).

Applying the conceptual model of MVE, Transitional Operational Linkages need to be established as a priority (Fig. 3). Operationalization via OFT requires addressing the issues of collaboration and logistics (Table 3). Although solutions such as building a temporary port off Gaza’s coast to increase and accelerate deliveries of humanitarian aid have been proposed, this solution is arguably not effective in terms of time, cost, and potential to be a military target. Given that at this time humanitarian food aid needs to come from international sources, Fig. 1 and Table 3 indicate that a solution lies in collaboration with military supply chains to effect delivery as an immediate priority, ideally as a peacekeeping force which circumvents the throttling of existing supply chains.

#### Ukraine

In the Russia–Ukraine war, the actions of military personnel are key to protecting agricultural production in support of food security. Drones are being utilized to supply frontline Ukrainian and Russian troops. Video footage indicates that the food and water is wrapped in cardboard and plastic to prevent damage from being dropped from the drone (e.g., Hambling 2024). This means of logistically supplying the frontlines is proving effective in spite of concerns of jamming or other interference leading to the loss of drones. Although there is potential for using drones in humanitarian supply chains, the role of technology in humanitarian response is focused on managing food safety and quality alongside being nutritious and culturally appropriate (Bounie et al. 2020), and evidence from downed drones indicates numerous shortcomings in these areas which could potentially compromise the abilities of warfighters.

Applying the conceptual model of MVE, given that drones are for now successfully delivering supplies, although there may be some efficiency concerns with damaged supplies and associated environmental impacts due to the packaging, these are relatively simple to remedy as opposed to other impacts of war on the environment. The focus needs to be on Diversity in Human Sustenance as a priority (Fig. 3). Operationalization via OFT requires addressing procurement, suppliers and collaboration. Having drones deliver canned food adds unnecessary weight compared to alternatives. The ready availability of these alternatives is a likely issue, which could be resolved from the perspective of time allocation via appropriate new logistical collaboration (forming Transitional Operational Linkages) to procure better food provisions from potential new suppliers to ensure adequate dietary breadth and nutritional content.

## Conclusion

The main contribution of this research is to present foraging supply chains as dynamic interconnected networks for sustainable, resilient, efficient, and equitable disaster food provisioning. The research question to which this study pertains has been addressed as follows: Foraging theory may be applied to disaster food provisioning using MVE as a lens to inform the fundamental connections among actors and their strategies. The key constituents of the related conceptual model are Diversity in Human Sustenance; Material and Process Efficiency; and Transitional Operational Linkages as shown in Fig. 3. For the MVE to be ecologically embedded, nutritional ecology, ecosystem sustainability, and ecological relationships need to be incorporated into its formation. Foraging theory enables operationalization of the conceptual model in terms of strategic goals, decision categories, and domains of choice, as shown in Table 3. Multiple failure points in food provisioning may be mitigated by adopting the strategies identified from the meta-ethnography cases.

### Limitations

The extended case study approach for meta-ethnography facilitates a more comprehensive theoretical explanation of MVE in the context of foraging theory, being both interpretive and critical, to strategically address strengths and weaknesses (Urrieta and Noblit 2018). The meta-ethnography followed Noblit and Hare’s (1988) well-established seven-stage process supported by an interdisciplinary and diverse research team. However, exclusion of studies not published in English presents a potential limitation of the synthesis findings. Similarly, although the included studies were from diverse countries, the results may not fully reflect all possible food supply provisioning experiences and strategies adopted globally.

## Data Availability

Data are available on request.
